# Bivalent Ligand UDCA-LPE Inhibits Pro-Fibrogenic Integrin Signalling by Inducing Lipid Raft-Mediated Internalization

**DOI:** 10.3390/ijms19103254

**Published:** 2018-10-20

**Authors:** Jie Su, Hongying Gan-Schreier, Benjamin Goeppert, Walee Chamulitrat, Wolfgang Stremmel, Anita Pathil

**Affiliations:** 1 Department of Internal Medicine IV, Gastroenterology and Hepatology, University of Heidelberg, Im Neuenheimer Feld 410, 69120 Heidelberg, Germany; suj@sustc.edu.cn (J.S.); Hongying.Gan-Schreier@med.uni-heidelberg.de (H.G.-S.); Walee.Chamulitrat@med.uni-heidelberg.de (W.C.); Wolfgang.Stremmel@med.uni-heidelberg.de (W.S.); 2 Department of Biology, Southern University of Science and Technology, Shenzhen 518055, China; 3 Institute of Pathology, University Hospital Heidelberg, Im Neuenheimer Feld 224, 69120 Heidelberg, Germany; Benjamin.Goeppert@med.uni-heidelberg.de

**Keywords:** integrin signalling, lipid raft-mediated internalization, hepatic fibrosis

## Abstract

Ursodeoxycholyl lysophosphatidylethanolamide (UDCA-LPE) is a synthetic bile acid-phospholipid conjugate with profound hepatoprotective and anti-fibrogenic functions in vitro and in vivo. Herein, we aimed to demonstrate the inhibitory effects of UDCA-LPE on pro-fibrogenic integrin signalling. UDCA-LPE treatment of human embryonic liver cell line CL48 and primary human hepatic stellate cells induced a non-classical internalization of integrin β1 resulting in dephosphorylation and inhibition of SRC and focal adhesion kinase (FAK). Signalling analyses suggested that UDCA-LPE may act as a heterobivalent ligand for integrins and lysophospholipid receptor1 (LPAR1) and co-immunoprecipitation demonstrated the bridging effect of UDCA-LPE on integrin β1 and LPAR1. The disruption of either the UDCA-moiety binding to integrins by RGD-containing peptide GRGDSP or the LPE-moiety binding to LPAR1 by LPAR1 antagonist Ki16425 reversed inhibitory functions of UDCA-LPE. The lack of inhibitory functions of UDCA-PE and UDCA-LPE derivatives (14:0 and 12:0, LPE-moiety containing shorter fatty acid chain) as well as the consistency of the translocation of UDCA-LPE and integrins, which co-fractionated with LPE but not UDCA, suggested that the observed UDCA-LPE-induced translocation of integrins was mediated by LPE endocytic transport pathway.

## 1. Introduction

Liver fibrosis is characterized by pathological accumulation of extracellular matrix (ECM). ECM is a collection of molecules which are secreted by cells and distributed in all organs and tissues consisting of collagens, proteoglycans, glycoproteins and glycosaminoglycans [1]. Although some other cell types in the liver can also contribute to fibrosis, it is generally accepted that activated hepatic stellate cells (HSC) are the main source of excessive ECM. Integrins are a family of heterodimeric transmembrane receptors composed of an α and a β subunit, which are involved in cell-cell and cell-matrix interactions. Binding of integrins to ECM components mediates the recruitment and activation of signalling proteins such as focal adhesion kinase (FAK) and SRC kinase, which play a central role in the transduction of intracellular integrin signalling events. Furthermore, integrins have been reported to be involved in HSC activation and migration [2] and found to be upregulated during liver fibrosis [3].

UDCA-LPE is a synthetic bile acid-phospholipid conjugate, which has exhibited profound hepatoprotective and anti-fibrogenic functions in vitro and in vivo [4,5]. The conjugate contains an ursodeoxycholic acid (UDCA) moiety, which by itself also exhibits protective effects against hydrophobic bile-acid-induced hepatocellular apoptosis in cholestatic liver disease [6] and has been approved for the treatment of primary biliary cirrhosis [7].

Notably, our former studies revealed that protective functions of UDCA-LPE are critically dependent on the conjugation between the bile acid and the phospholipid whereas the individual compounds UDCA or LPE showed only little efficacy in different in vitro [4] and in vivo models [8]. These results imply that the conjugation due to its hydrophobicity is decisive in order to facilitate the interaction of UDCA-LPE with lipid membranes [9,10] rendering it a promising drug candidate for membrane lipid therapy [11].

Herein, we demonstrate the interaction of UDCA-LPE with integrins leading to integrin internalization via lipid rafts and subsequent inhibition of fibrogenic signalling. These events represent a novel mechanism of UDCA-LPE in support of its potent anti-fibrogenic effects previously observed in experimental mouse models of liver disease [5].

## 2. Results

### 2.1. UDCA-LPE Induces Translocation of Integrins

**Figure 1:**
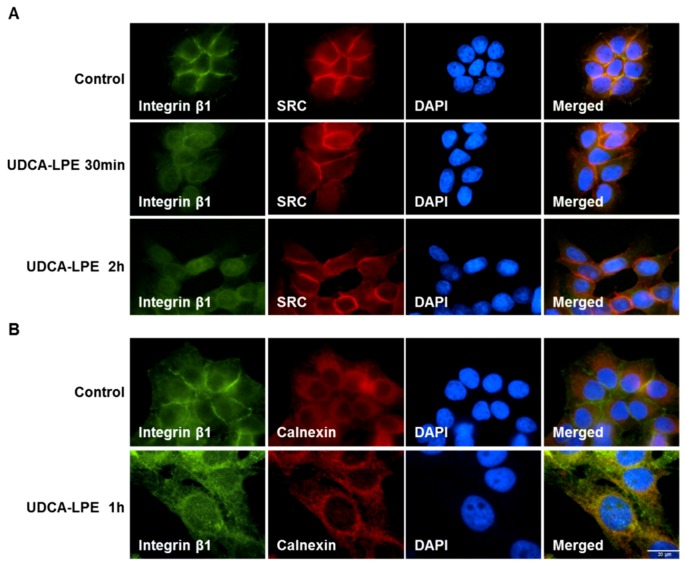
UDCA-LPE modulates the localization of integrin β1. Representative fluorescence microscopy images of CL48 cells after treatment with 90 μM UDCA-LPE for (**A**) 30 min or 2 h and (**B**) 1 h. Immunofluorescence showed the staining of (**A**) integrin β1 (green), SRC (red) and DAPI (blue) and (**B**) integrin β1 (green), calnexin (red) and DAPI (blue). DAPI was used for nuclear staining.

The interaction with ECM leads to an autophosphorylation of FAK at Tyr397 with subsequent binding of FAK to SRC, which in turn activates SRC leading to phosphorylation of FAK at Tyr576/577 and Tyr925, which is known to be essential for its kinase activity [12]. After phosphorylation in response to integrin engagement, FAK and SRC trigger pro-fibrogenic signalling both in vivo and in vitro [13,14]. As non-kinase receptors, integrins activate FAK by a conformational change. Thus, the co-localization and interaction between integrins and FAK/SRC are considered to be essential for proper signalling. In the absence of UDCA-LPE, integrin β1 and SRC were found to be localized predominantly at cell-to-cell contacts of CL48 liver cells (Figure 1A). Upon addition of UDCA-LPE for 30 min, most of integrin β1 migrated away from plasma membrane while SRC localization was not affected (Figure 1A). After 2 h treatment, integrin β1 accumulated more pronouncedly at the nuclear envelope (Figure 1A). Despite of this integrin β1 translocation, the localization of active FAK (pFAK Tyr397) at the focal adhesions of CL48 cells was not affected by UDCA-LPE (Figure S1). UDCA-LPE-induced internalization of integrin β1 was also observed in HHStec cells (Figure S2A). The co-localization of integrin β1 and the endoplasmic reticulum (ER) marker calnexin in CL48 cells (Figure 1B) and HHStec cells (Figure S2B) upon UDCA-LPE treatment suggests the localization of endocytosed integrin β1 at the ER. Notably, treatment of CL48 cells with UDCA, LPE or UDCA + LPE had no effect on integrin β1 localization (Figure S3). Besides integrin β1, UDCA-LPE similarly induced the translocation of other integrins including integrin α2, α3, α5, αv, β4 and β5 (Figure S4). In the absence of UDCA-LPE, these integrins displayed some differences in terms of localization, that is, integrins α2, α3 and α5 localized at plasma membrane, integrin αv at the cytoplasm and integrin β5 at focal adhesions (Figure S5). However, the internalization of these integrins by UDCA-LPE was similar to that of integrin β1.

### 2.2. Integrin Translocation by UDCA-LPE Suppresses FAK and SRC Phosphorylation

**Figure 2:**
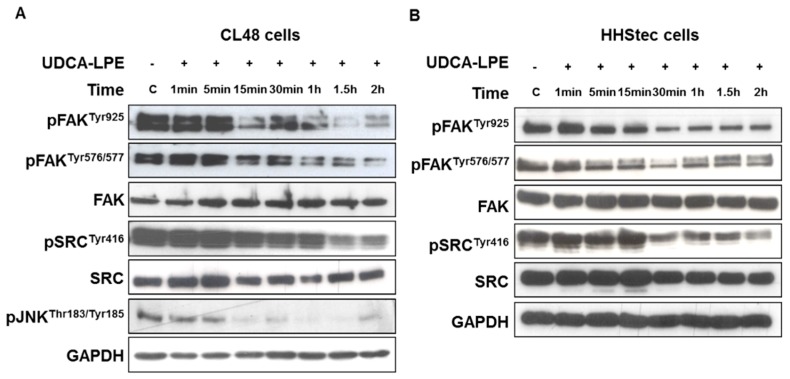
UDCA-LPE inhibits phosphorylation of SRC and FAK. (**A**) CL48 cells and (**B**) HHStec cells were treated with 90 μM UDCA-LPE for 1 min to 2 h. Cell lysates were probed with antibodies against phospho-FAK (Tyr925), phospho-FAK (Tyr576/577), FAK, phospho-SRC (Tyr416), SRC and phospho-JNK (Thr183/Tyr185). GAPDH was used as control for equal protein loading.

Translocation of integrin β1 with subsequent loss of its co-localization with SRC (Figure 1A) was associated with decreased phosphorylation of FAK (Tyr925 and Tyr576/577) and SRC (Tyr416) upon UDCA-LPE treatment of CL48 (Figure 2A) and HHStec cells (Figure 2B) in a time-dependent manner from 15 min to 2 h. In CL48 cells, phosphorylation of c-Jun N-terminal kinases (JNK) which is a downstream target protein of FAK was also decreased by UDCA-LPE treatment (Figure 2A). Thus, UDCA-LPE inhibited integrin signalling after induction of integrin internalization via an inhibition of FAK and SRC phosphorylation.

### 2.3. RGD-Containing Peptide GRGDSP Inhibits UDCA-LPE-Induced Translocation of Integrins

**Figure 3:**
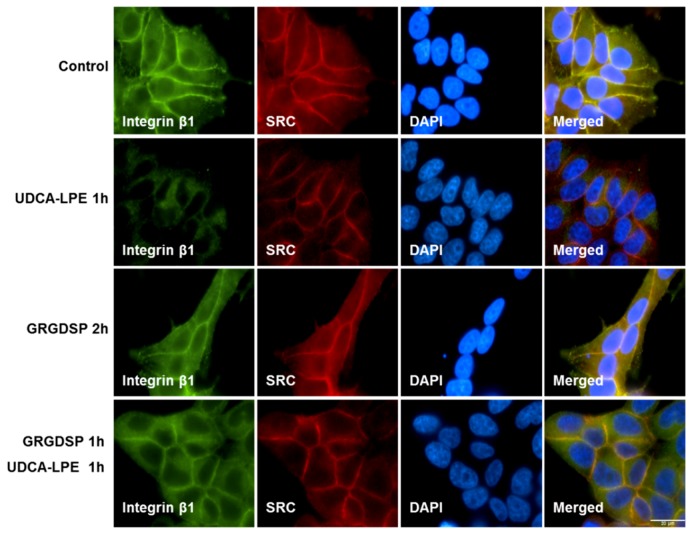
RGD-containing peptide GRGDSP inhibits UDCA-LPE–induced translocation of integrins. Representative fluorescence microscopy images of CL48 cells after treatment with 200 μg/mL RGD-containing peptide GRGDSP for 1 h and 90 μM UDCA-LPE for additional 1 h. IF staining of integrin β1 (green), SRC (red) and DAPI (blue). DAPI was used for nuclear staining.

The most prevalent integrin recognition site present in ECM contains a tripeptide motif composed of L-arginine, glycine and L-aspartic acid (RGD). RGD-containing peptides, which bind to the RGD-recognition site of integrin, inhibit their binding to the ECM. Although UDCA-LPE mediated the translocation of multiple integrins, it did not induce the translocation of integrin α1 as observed in CL48 cells (Figure S5). Integrin α1 can uniquely form a α1β1 heterodimer, which unlike most other integrins does not recognize RGD motif in ECM [15]. The lack of integrin α1 translocation implies that UDCA-LPE-induced translocation of integrins may solely depend on the RGD-recognition motif. GRGDSP peptide which blocks the RGD-recognition motif in integrins was therefore used to disrupt the binding of integrins to the RGD motif in ECM. GRGDSP alone had no effect on integrin localization (Figure 3). However, pre-treatment with GRGDSP markedly blocked UDCA-LPE-induced translocation of integrin β1 (Figure 3).

### 2.4. UDCA-LPE Binds to Integrins with Its UDCA-Moiety

**Figure 4:**
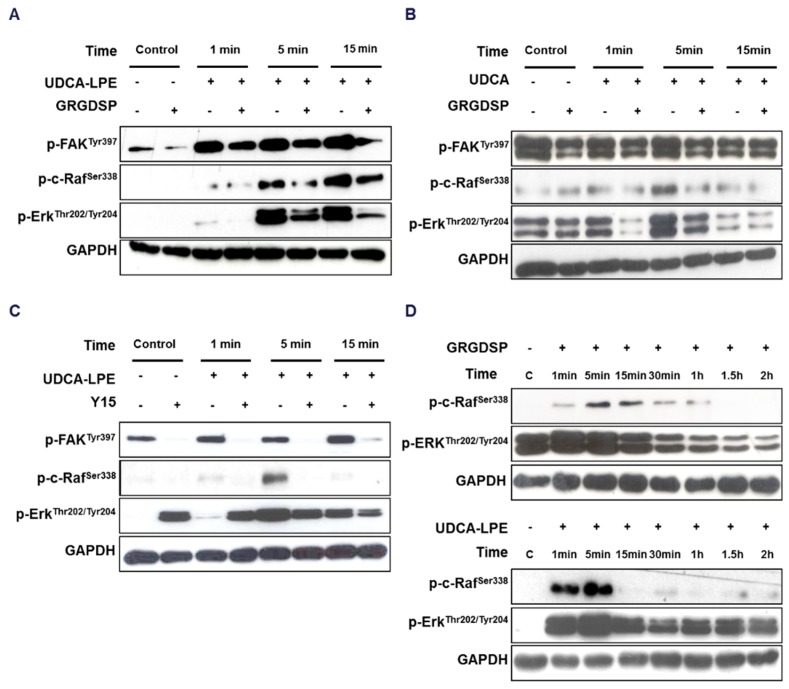
UDCA-LPE and UDCA induce integrin-dependent phosphorylation of c-Raf and ERK. (**A**–**C**) CL48 cells were treated with (**A**,**B**) 100 μg/mL RGD containing peptide GRGDSP or (**C**) 100 μM FAK inhibitor 1,2,4,5-benzenetetraamine tetrahydrochloride (Y15) for 30 min and (**A**,**C**) 90 μM UDCA-LPE or (**B**) 90 μM UDCA for 1 to 15 min. Lysates were probed with antibodies against phospho-FAK (Tyr397), phospho-c-Raf (Ser338) and phospho-ERK 1/2 (Thr202/Tyr204). (**D**) CL48 cells were treated with 100 μg/mL RGD peptide or 90 μM UDCA-LPE for 1 min to 2 h. Lysates were probed with antibodies against phospho-c-Raf (Ser338) and phospho-ERK 1/2 (Thr202/Tyr204). GAPDH was used as control for equal protein loading.

It is known that activation of integrin signalling involves autophosphorylation of FAK at Tyr397, which leads to an interaction of FAK with SRC [12]. With a short incubation time of 1–5 min, UDCA-LPE stimulated the phosphorylation of FAK (Tyr397) as well as the downstream targets c-Raf (p-Ser338) and ERK (p-Thr202/Tyr204) (Figure 4A). This activation was inhibited by GRGDSP pre-treatment. Phosphorylation of FAK at Tyr397, c-Raf and ERK was also observed by UDCA treatment (Figure 4B). Similar to GRGDSP, pre-treatment with FAK inhibitor 1,2,4,5-benzenetetraamine tetrahydrochloride (Y15) significantly blocked UDCA-LPE-induced phosphorylation of FAK (Tyr397), c-Raf and ERK (Figure 4C), suggesting a FAK-dependent mechanism. We found that RGD peptide alone also induced the phosphorylation of c-Raf and ERK after 1-5 min treatment time (Figure 4D) in a similar manner as UDCA-LPE (Figure 4D) and UDCA (Figure 4B). This suggested that these compounds triggered integrin signalling in a similar manner like RGD peptide. Taken together, our results suggest that an interaction of UDCA-LPE with integrins may employ the UDCA-moiety of the molecule. Further binding experiments have to prove this hypothesis.

### 2.5. UDCA-LPE Serves as a Bivalent Ligand Bridging Between Integrin β1 and LPAR1

**Figure 5:**
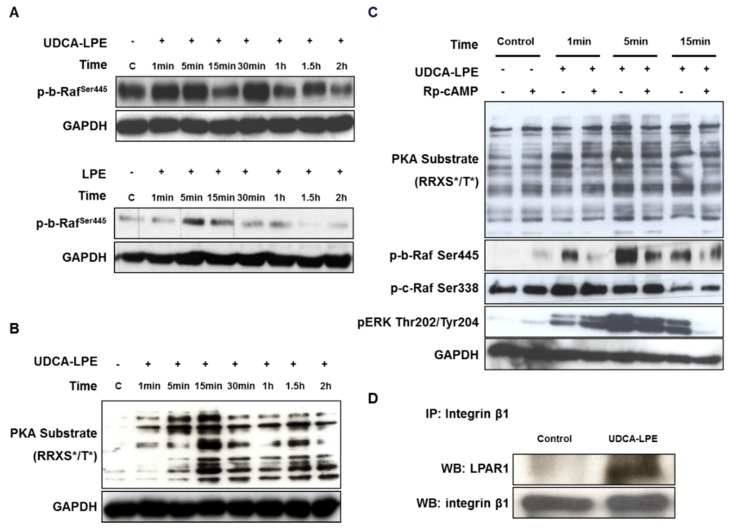
UDCA-LPE induces the LPE signalling and complex formation between LPAR1 and integrin β1. (**A**,**B**) CL48 cells were treated with (**A**,**B**) 90 μM UDCA-LPE or (**A**) 90 μM LPE for 1 min to 2 h. Lysates were probed with antibodies against (**A**) phospho-b-Raf (Ser445) and (**B**) PKA substrates (RRXS*/T*). (**C**) CL48 cells were treated with 200 μM Rp-cAMP for 30 min and 90 μM UDCA-LPE for 1 min to 15 min. Lysates were probed with antibodies against PKA substrate (RRXS */T *), phospho-b-Raf (Ser445), phospho-c-Raf (Ser338) and phospho-ERK (Thr202/Tyr204). (**D**) CL48 cells were treated with 90 μM UDCA-LPE for 1 h. Integrin β1-containing proteins were immunoprecipitated with a polyclonal anti-integrin β1 antibody and immunoblotted using anti-LPAR1 or anti-integrin β1 antibody.

We found that treatment of CL48 cells with UDCA-LPE or LPE was able to induce phosphorylation of b-Raf at Ser445 in the first 15 min (Figure 5A). It is known that LPE interacts with a G protein-coupled receptor LPAR1 [16] and that LPAR activation induces the activation of PKA [17,18]. We used anti-PKA substrates (RRXS*/T*) antibody to determine the activity of PKA. UDCA-LPE was able to rapidly induce phosphorylation of PKA substrates maximizing at 15 min (Figure 5B). The UDCA-LPE-induced activation of b-Raf (but not c-Raf) and ERK was inhibited by pre-treatment with PKA antagonist Rp-cAMP (Figure 5C). These data showed the ability of UDCA-LPE to trigger LPE/LPAR1 signalling via PKA/b-Raf/ERK pathways. We further performed immunoprecipitation of integrin β1 followed by immunoblotting with an anti-LPAR1 antibody. LPAR1 was nearly undetectable in the pull-downs of control cells whereas LPAR1 protein levels were markedly elevated in those of UDCA-LPE-treated cells (Figure 5D). Our results suggest that UDCA-LPE may act as a bivalent ligand bridging between integrins and LPAR1 to form a tri-component complex.

### 2.6. LPE-Moiety is Necessary for UDCA-LPE-Induced Translocation of Integrin β1 and Suppressed FAK and SRC Phosphorylation

**Figure 6:**
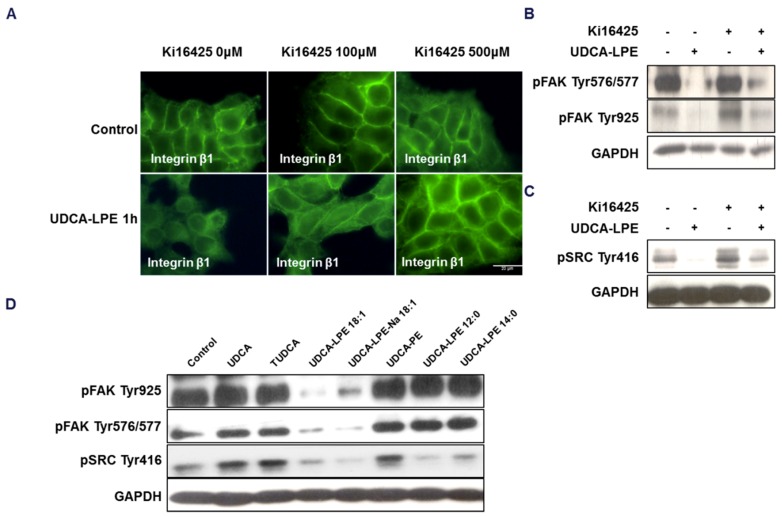
Translocation of integrin β1 and dephosphorylation of SRC and FAK is dependent on the LPE moiety of UDCA-LPE. (**A**) Representative fluorescence microscopy images of CL48 cells after treatment with LPAR antagonist Ki16425 at 100 μM or 500 μM for 1 h and 90 μM UDCA-LPE for additional 1 h. IF of anti-integrin β1 (green). (**B**) CL48 cells were treated with 50 μM Ki16425 for 1 h, followed with 90 μM UDCA-LPE for additional 1h. Lysates were probed with antibodies against phospho-FAK (Tyr925) and phospho-FAK (Tyr576/577). (**C**) CL48 cells were treated with 1mM Ki16425 for 1 h, followed with 90 μM UDCA-LPE for additional 1 h. Lysates were probed with antibodies against phospho-SRC (Tyr416). (**D**) CL48 cells were treated with 90 μM UDCA, TUDCA, UDCA-LPE, UDCA-LPE-Na, UDCA-PE, UDCA-LPE 12:0 or UDCA-LPE 14:0 for 2 h. Lysates were probed with antibodies against phospho-FAK (Tyr925) and phospho-FAK (Tyr576/577). GAPDH was used as control for equal protein loading.

To dissect the role of LPAR1, we further utilized an LPAR antagonist Ki16425, which was reported to disrupt the binding of LPE to LPAR1 [16]. We found that Ki16425 pre-treatment significantly blocked UDCA-LPE-induced translocation of integrin β1 in a concentration-dependent manner (Figure 6A). Additionally, UDCA-LPE-induced inhibition of phosphorylation of FAK (Tyr576/577 and Tyr925) (Figure 6B) and SRC (Tyr416) (Figure 6C) was nearly completely abolished by pre-incubation with Ki16425. It has been reported that the activity of lysophosphatidic acids to bind and activate LPAR decreases with a shorter fatty-acid chain length [19,20]. Thus, we treated CL48 cells with UDCA-PE (a conjugate of UDCA and 18:1, 18:1 PE), UDCA-LPE (12:0) (UDCA conjugated with 12:0 LPE) or UDCA-LPE (14:0) (UDCA conjugated with 14:0 LPE). Unlike UDCA-LPE (UDCA conjugated with 18:1 LPE), UDCA-PE, UDCA-LPE (12:0) or UDCA-LPE (14:0) did not decrease but rather slightly increase the phosphorylation of FAK (Tyr 925 and Tyr576/577) and SRC (Tyr416), which was found to be similar to UDCA and tauro-UDCA (TUDCA) (Figure 6D). Taken together, our results suggested that the LPE-moiety and its association with LPAR1 were essential for UDCA-LPE-induced translocation of integrin β1 and inhibition of SRC and FAK phosphorylation.

### 2.7. UDCA-LPE Mediates the Compartmentalization of Integrins into Lipid Rafts

**Figure 7:**
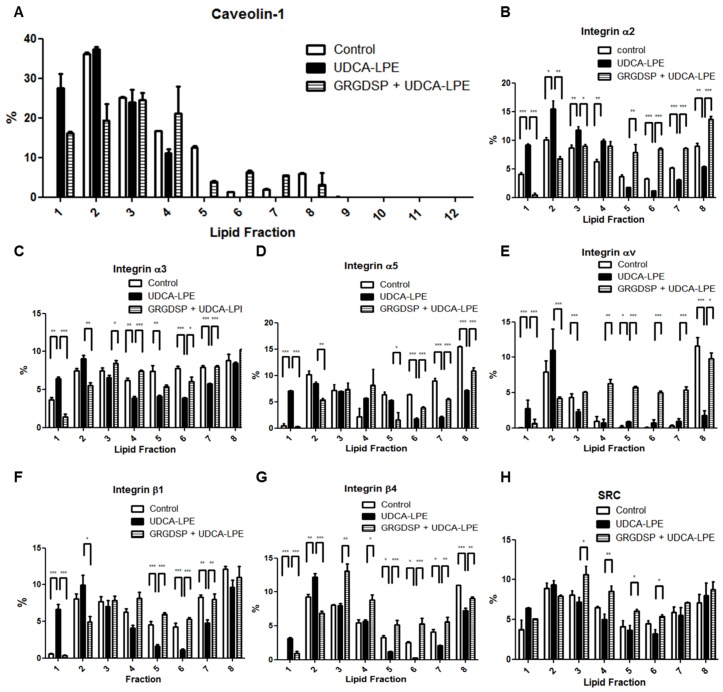
UDCA-LPE mediates compartmentalization of integrins into lipid rafts. (**A**–**C**) Lipid fractionation of CL48 cells after treatment with 200 μg/mL RGD-containing peptide GRGDSP for 1 h and 90 μM UDCA-LPE for 30 min. Separated fractions were immunoblotted with antibodies against (**A**) caveolin-1, (**B**) integrin α2, (**C**) integrin α3, (**D**) integrin α5, (**E**) integrin αv, (**F**) integrin β1, (**G**) integrin β4 or (**H**) SRC respectively. The protein of interest was normalized to the amount of all proteins in 12 fractions as 100%. Data are means ± the standard deviation of three independent experiments. *** *p* < 0.001, ** *p* < 0.01, * *p* < 0.05.

Cell lysates were subjected to lipid fractionation and the levels of various integrins in 12 fractions were analysed by western blotting. A marker for lipid rafts caveolin-1 was mostly detected in fractions 2–4 of control CL48 cell lysates and in fractions 1–4 in cells treated with UDCA-LPE for 30 min (Figure 7A). This indicated that the integrity of lipid rafts was not disturbed by UDCA-LPE and that lipid rafts were maintained in lower density fractions 1–4. UDCA-LPE treatment did not alter SRC protein concentrations in any of lipid fractions (Figure 7H) but markedly increased concentrations of integrin α2, α3, α5, αv, β1 and β4 in lipid-raft fractions 1–4 concomitant with decreased concentrations in fractions 5–8 (Figure 7B–G). Moreover, co-incubation with GRGDSP inhibited UDCA-LPE-induced translocation of these integrins to lipid-raft fractions (Figure 7B–G).

### 2.8. Integrin-Bound UDCA-LPE Translocated into Lipid Rafts, Which Co-Fractionated with LPE but Not UDCA

**Figure 8:**
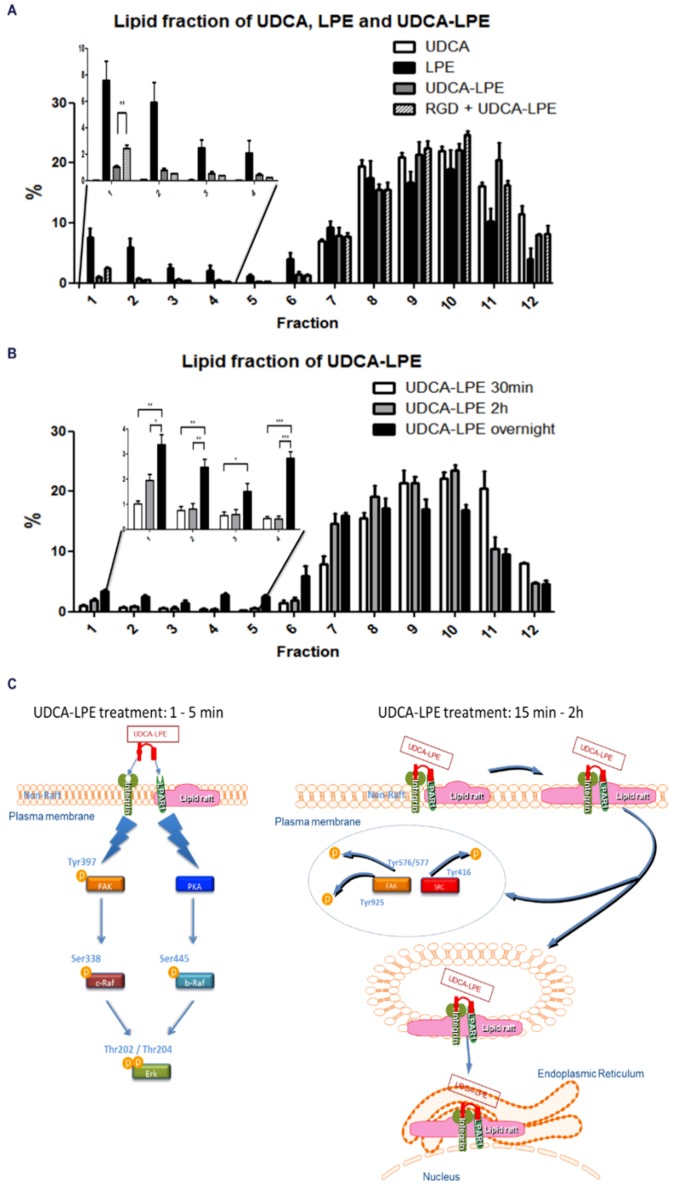
Distribution of UDCA, LPE and UDCA-LPE in lipid fractions. (**A**,**B**) Lipid-raft fractionation of CL48 cells after treatment with (**A**) 90 μM UDCA, 90 μM LPE, 90 μM UDCA-LPE for 30 min or 200 μg/mL GRGDSP for 1 h and 90 μM UDCA-LPE for additional 30 min or (**B**) 90 μM UDCA-LPE for 30 min, 2 h or overnight. Separated fractions were subjected to liquid-chromatography mass spectrometry for quantification of UDCA, LPE or UDCA-LPE in UDCA, LPE or UDCA-LPE-treated cells, respectively. In each treated group, the total levels of UDCA, LPE or UDCA-LPE in 12 fractions was normalized as 100%. Data are means ± the standard deviation of three independent experiments. *** *p* < 0.001, ** *p* < 0.01, * *p* < 0.05. (**C**) Schematic time-dependent model for anti-fibrogenic effects of UDCA-LPE. The lightning graphic means stimulation: the binding of UDCA-LPE with integrin activates phosphorylation of FAK and SRC. The arrows mean (1) translocation of the UDCA-LPE complex into lipid rafts, which (2) results in the dephosphorylation of FAK and SRC.

The intracellular transport of a heterobivalent ligand could be determined by one of its receptors [21]. To investigate which receptor determined the localization of UDCA-LPE, we treated CL48 cells with UDCA, LPE or UDCA-LPE for 30 min and cell lysates were subjected to lipid-raft fractionation and the concentrations of UDCA, LPE or UDCA-LPE in 12 fractions were respectively determined by high-performance liquid chromatography-tandem mass spectrometry. UDCA was localized only in non-raft fractions, whereas LPE was present in both raft- and non-raft fractions (Figure 8A), suggesting that UDCA receptors were localized only in non-raft fractions whereas LPE receptors were present in both fractions. UDCA-LPE displayed an integrated localization of both UDCA and LPE and the proportion of UDCA-LPE in raft fractions 1–4 was in parallel to that of LPE (Figure 8A, Inset) suggesting that the initial localization of UDCA-LPE was determined by both UDCA- and LPE-receptors. GRGDSP, which inhibited the binding of UDCA-LPE to integrins, decreased the proportion of UDCA-LPE in non-raft fractions and increased the proportion in lipid-raft fractions, indicating that integrin-bound UDCA-LPE was initially localized in non-raft fractions. After incubation with UDCA-LPE for 2 h or overnight an increased proportion of UDCA-LPE was detected in lipid rafts in a time-dependent manner (Figure 8B), suggesting a translocation of integrin-bound UDCA-LPE to lipid rafts at a longer incubation time. These data were consistent with the translocation of integrins into lipid rafts by UDCA-LPE treatment (Figure 7), suggesting a co-translocation of integrins with UDCA-LPE. Taken together, the co-translocation of integrins and UDCA-LPE was determined by LPE receptors.

## 3. Discussion

As effective therapeutic options against liver fibrosis are limited to date, the proposal of novel compounds which target pro-fibrogenic pathways is urgently needed. The bile acid-phospholipid conjugate UDCA-LPE has been proven to exhibit potent anti-fibrogenic functions in vitro and in vivo [5]. In this study, we analysed enforced translocation of integrins by UDCA-LPE as a possible mechanism for its anti-fibrogenic effects. We showed that UDCA-LPE can associate to the RGD-recognition motif in integrins and LPAR1 with its UDCA- and LPE-moiety, respectively. The latter binding acts as a transporter of UDCA-LPE into lipid-rafts occurring simultaneously with an internalization of UDCA-LPE-bound integrins to the ER and the nuclear envelope. The subsequent loss of SRC co-localization with integrins decreased phosphorylation levels of SRC and FAK leading to an inhibition of pro-fibrogenic activity.

Recent studies have reported that TUDCA stimulates integrin-dependent phosphorylation of SRC, FAK, ERK and p38MAPK [22,23]. Similar to TUDCA, UDCA and UDCA-LPE stimulated integrin- and FAK-dependent c-Raf and ERK phosphorylation in CL48 cells as well (Figure 4). Interestingly, recent results using a 3D model of integrin α5β1 have shown the importance of the RGD-recognition motif as a sensor of TUDCA. However, TUDCA has an intracellular effect on integrin α5β1 rather than at the plasma membrane [24]. As UDCA and TUDCA are known to be located at the interfacial outer surface of plasma membrane [25,26], this may be the case for the binding of UDCA-LPE to the extracellular domain of integrins as we showed that UDCA-LPE was able to induce integrin internalization at plasma membrane (Figure 1) and that GRGDSP could inhibit the translocation of integrins (Figure 2B–D and Figure 3).

The design of heterobivalent ligands to target two different receptors has previously been used for pharmacological purposes [27]. As a novel heterobivalent ligand (Figure 8C), UDCA-LPE was not only able to bind to integrins with its UDCA-moiety but also triggered LPE/LPAR1 signalling through its LPE-moiety (Figure 5A–C). The stimulation of UDCA and LPE signalling occurred in the first 5 min of UDCA-LPE treatment by association of UDCA-LPE with integrins and LPAR1 (Figure 8, left). This process may be equivalent to UDCA + LPE treatment. However, the character of UDCA-LPE to bridge integrins and LPE receptors, which was confirmed by co-immunoprecipitation of integrins and LPAR1 (Figure 5D), rendered UDCA-LPE to have a specific function in pulling integrins into the intracellular transport pathway of LPE. We hypothesize that this results in the translocation of integrins from the plasma membrane to the ER and the nuclear envelop observed at a longer incubation time (Figure 8, right). Additional experiments have to further evaluate the localization and intracellular trafficking of endocytosed integrins. Our results showed that LPE alone had no effect on the localization of integrins and that UDCA-LPE-induced translocation of integrins and the inhibition of integrin signalling were dependent on the LPE-moiety of UDCA-LPE.

It has been reported that LPAR1 is localized partially in lipid rafts [28] and that disruption of lipid rafts impairs the function of LPAR1 [29,30]. As LPAR1 is a receptor of LPE [16], we also found that ~18% of LPE was localized in lipid rafts upon LPE treatment for 30 min (Figure 8A). Although UDCA has been reported to antagonize the deoxycholate-induced cholesterol depletion [31], it has been shown that UDCA owns a much higher affinity to non-raft than to lipid-raft fractions [25]. Consistent with this, almost no UDCA was detectable in raft fractions of UDCA-treated cells (Figure 8A). The localization of integrins in non-rafts and LPE endocytic transport pathway destined in lipid-rafts indeed allowed an opportunity for UDCA-LPE to be the mediator for integrin translocation (Figure 8C). UDCA-LPE was translocated into lipid rafts via LPE/LPAR1 axis (Figure 8) concomitant with its internalization (Figure 7) via UDCA/integrin axis.

The general mechanism for endocytic transport of LPE has not been well understood. LPARs are normally localized in both clathrin and caveolar endocytic microdomains and the latter is thought to respond to LPAR internalization because of LPAR co-localization with caveolin-1 in the nucleus [28]. It has been shown that LPAR-induced gene expression is insensitive to caveolea-disrupting agents filipin and methyl-β-cyclodextrin [28], suggesting that LPAR internalization does not necessarily rely on the structure of caveolea. Our data also supported this notion as filipin or methyl-β-cyclodextrin treatment did not inhibit integrin translocation induced by UDCA-LPE (data not shown). The independency from caveolea was one of the features of UDCA-LPE-induced internalization of integrins which may be different from the previously reported integrin endocytosis/recycling pathway [32].

Integrins cross-talk with crucial pro-fibrogenic pathways such as TGFβ1 and PDGF signalling [33] and are therefore regarded as attractive therapeutic targets for the treatment of fibrotic disease. Most inhibitors of integrins including antibodies and cyclic RGD-containing peptides [34,35] have focused on the inhibition of integrin-induced cell-to-ECM and cell-to-cell interactions. However, the use of RGD peptides for fibrosis treatment is quite limited [36,37] because of their lack of persistent effects [38]. Due to multiple binding sites of integrins for ECM [39], an exclusive blockade of RGD-recognition motif may not completely disrupt the binding of integrins to ECM. Here, we could demonstrate that UDCA-LPE not only occupied the RGD-binding sites in integrins but also induced integrin internalization which completely disrupted the ECM-binding to integrins at the plasma membrane (Figure 8C). Thus, UDCA-LPE emerged as an effective inhibitor of RGD-binding integrins more potent than the typical RGD-containing peptide.

It is well-recognized that integrin-induced signalling plays a crucial role in fibrogenesis and that the downstream proteins FAK and SRC play an essential during pro-fibrotic signalling [40,41]. RGD peptide has been reported to activate integrins [42,43], which may also promote fibrogenic signalling. Our data supported this notion as we found that RGD peptide was able to induce integrin signalling (Figure 4D). Unlike RGD peptide, by removing the activator of FAK and SRC UDCA-LPE treatment led to persistent inhibition of integrin signalling after long incubation of CL48 cells and HHStec cells (Figure 2 and Figure 8C, right) thus displaying a very potent anti-fibrogenic effect.

In present study, we demonstrated a possible novel pharmacological tool for integrin inhibition, where UDCA-LPE did not function as a direct inhibitor of integrins per se but as a heterobivalent ligand bridging between integrins and LPAR1. By the action of LPE/LPAR1 transporters in cells, UDCA-LPE was able to induce the translocation of integrins leading to a loss of co-localization with SRC, which resulted in dephosphorylation of FAK and SRC and inhibition of downstream fibrogenic targets. This elucidated mechanism of action renders UDCA-LPE as a drug candidate for the treatment of liver fibrosis.

## 4. Materials and Methods

### 4.1. Reagents and Cell Culture

All reagents as well as the cultures and treatment of human embryonic liver CL48 cell line and Human Hepatic Stellate Cells (HHStec) are shown in Supplementary Materials.

### 4.2. Western Blotting

Lysates of treated cells were subjected to western blotting analysis (Supplementary Materials).

### 4.3. Immunofluorescence

Paraformaldehyde-fixed cells were subjected to immunofluorescence (Supplementary Materials).

### 4.4. Lipid Fractionation

For each treatment group, CL48 cells were cultured in 20 × 75 cm^2^ culture flasks. After UDCA, LPE or UDCA-LPE treatment, cells were rinsed with PBS and scraped into 10 mL buffer containing 2 mM HEPES, 150 mM NaCl, 1 mM EGTA, 5 mM sodium vanadate, 10 mM sodium azide, 10 mM sodium pyrophosphate, 100 μg/mL PMSF, 1 mM sodium orthovanadate and 10 μl/mL protease inhibitor cocktail. Cells were homogenized and the lysates were centrifuged at 800× *g* at 4 °C for 10 min. Two mL of supernatants were incubated at 37 °C for 4 min and then incubated with 0.02 g Brij 98 at 37 °C for 5 min. The extracts were adjusted to 4 mL with 2 M Sucrose and cooled down in ice for 1 h. The extracts were gently overlaid with successive decreasing sucrose densities solutions (0.9–0.8–0.75–0.7–0.6–0.5–0.4–0.2 mol/L Sucrose) to prepare a discontinuous sucrose gradient. The gradients were centrifuged at 200,000× *g* in a Beckman SW 41Ti rotor for 22 h at 4 °C. Twelve fractions (1 mL for each fraction) were collected and used for western blotting and liquid-chromatography mass spectrometry (LC/MS-MS) analyses. The concentrations of total targets (proteins or lipids) in 12 fractions were normalized to 100% and the proportion or abundance of each target was reported in %. Cell lysates were subjected to sucrose density-gradient centrifugation for lipid fractionation.

### 4.5. Quantification of UDCA-LPE, UDCA and LPE

Following lipid fractionation, 500 μL of each fraction were extracted with 3 mL chloroform-methanol 2:1 mixture, 500 μL water and 20 μL internal standard D4-UDCA. Following centrifugation at 2500 rpm for 5 min, the lower chloroform phase was collected. Three mL of 2:1 chloroform-methanol mixture was added to the upper phase, extracted the second time and again centrifuged at 2500 rpm for 5 min. The lower phase was collected, combined with the previous chloroform phase and added to 0.4 mL 50 mM citric acid. Following mixing and centrifugation the lower phase was collected in a glass tube and the solvent was evaporated to dryness. The dried lipids were dissolved in 180 μL methanol. Concentrations of UDCA-LPE, UDCA and LPE in each lipid fraction were quantified using a liquid-chromatography mass spectrometer. The responses were calculated from the ratio of UDCA-LPE, UDCA, or LPE peak and D4-UDCA. Concentrations in nmol/mg protein were calculated from response of UDCA-LPE, UDCA and LPE used in standard curves. LC/MS-MS machine and running conditions are described in our published work [44]. Briefly, the separation was achieved by using a Phenomenex Luna C18 (Phenomenex, Aschaffenburg, Germany) column (100 × 2.0 mm, 3 μm) fitted on a separation module of a Waters 2695 (Waters, Milford, MA, USA). Binary solvents were 80% H2O/MeOH with 8 mM ammonium acetate, pH 8.0 (solvent A) and 95% MeOH/H_2_O with 8 mM ammonium acetate, pH 8.0 (solvent B). The flow rate was maintained at 0.2 mL/min and the gradient was started with 100% solvent A for 2.5 min, changed to 100% solvent B in 1 min, held for 16.5 min and returned to the initial condition in 3 min. Separated fractions were detected on-line by an electrospray ionization source of the tandem mass spectrometer (Quattro micro API, Micromass Waters, Waters, Milford, MA, USA).

### 4.6. Immunoprecipitation

Lysates of treated cells were subjected to immunoprecipitation analysis (Supplementary Materials).

### 4.7. Statistical Analysis

Statistical analysis was performed using Prism Software version 4.0 (GraphPad, La Jolla, San Diego, CA, USA).

Please see Supplementary Materials for detailed information.

## 5. Conclusions

UDCA-LPE enforces internalization of integrins leading to an inhibition of downstream signalling pathways. As a possible novel mode of integrin inhibition, we described the simultaneous bivalent ligation of integrins and LPAR1 by via the LPE endocytic transport pathway. Thus, UDCA-LPE emerges as drug candidate for treatment of liver fibrosis by inhibiting integrin signalling via its internalization.

## Supplementary Materials

Supplementary materials can be found at http://www.mdpi.com/1422-0067/19/10/3254/s1.

## Abbreviations

ECM	extracellular matrix
ER	endoplasmic reticulum
ERK	extracellular signal-regulated kinase
FAK	focal adhesion kinase
HHStec	primary human hepatic stellate cells
LPAR1	lysophosphatidic acid receptor 1
LPE	lysophosphatidylethanolamine
RGD	L-arginine, glycine and L-aspartic acid
TUDCA	tauro-ursodeoxycholic acid
UDCA	ursodeoxycholic acid
UDCA-LPE	ursodeoxycholyl lysophosphatidylethanolamide
