# Supplementary material for: Bivalent Ligand UDCA-LPE Inhibits Pro-Fibrogenic Integrin Signalling by Inducing Lipid Raft-Mediated Internalization
Source: Int J Mol Sci. 2018 Oct 20;19(10):3254. doi: 10.3390/ijms19103254 (PMC6214129; doi:10.3390/ijms19103254)
Supplement: Supplementary file 1 [file ijms-19-03254-s001.zip › ijms-360373-SI.pdf]

Int. J. Mol. Sci. 2018, 19, 3254; doi:10.3390/ijms19103254 www.mdpi.com/journal/ijms

International Journal of *Molecular Sciences*

Bivalent Ligand UDCA-LPE Inhibits Pro-Fibrogenic Integrin Signaling by Inducing Lipid raft-mediated Internalization

Supplementary Methods

Reagents. Custom synthesis of UDCA-LPE was performed by ChemCon (Freiburg, Germany). UDCA-PE was custom synthesized by Biomol (Hamburg, Germany). RGD-containing peptide GRGDSP was purchased from Bachem (Weil, Germany). UDCA, 3-sn-lysophosphatidyl ethanolamine, FAK inhibitor 1,2,4,5-benzenetetraamine tetrahydrochloride (Y15) and LPAR antagonist Ki16425 were purchased from Sigma-Aldrich (Taufkirchen, Germany). Antibodies against integrin α2, α5, αv, β1 and β 3 were purchased from Abcam (Cambridge, Great Britain). Antibodies against integrin α1, α3, β4, β5, β6, GAPDH, β-actin were purchased from Santa Cruz Biotechnology (Heidelberg, Germany). Antibodies against SRC, phospho-SRC (Tyr416), FAK, phospho-FAK (Tyr397), phospho-FAK (Tyr576/577), phospho-FAK (Tyr925), phospho-c-Raf (Ser338), phospho-b-Raf (Ser445), phospho-ERK (Thr202/Tyr204), phospho-SAPK/JNK (Thr183/Tyr185) and calnexin were purchased from Cell Signaling Technology (Frankfurt, Germany). Anti-LPAR1 antibody was purchased from Biozol (Eching, Germany). UDCA-LPE derivatives [UDCA-LPE (12:0) and UDCA-LPE (14:0)] were kind gifts from Phenex Pharmaceuticals AG (Heidelberg, Germany). All other chemicals were purchased from Sigma-Aldrich (Taufkirchen, Germany) unless stated otherwise.

Cell culture. Human embryonic liver cell line (CL48) was obtained from American Type Culture Collection (Manassas, VA, USA) and cultured in DMEM (Life Technologies, Darmstadt, Germany) supplemented with 10% fetal bovine serum (FBS) (PAA Laboratories GmbH, Freiburg, Germany), 2 mM L-glutamine, MEM non-essential amino acids, 100 U/L penicillin, and 100 g/L streptomycin at 37°C and 5.1% CO2. Human Hepatic Stellate Cells (HHStec) were purchased from Innoprot (Biscay, Spain, Catalog# P10653) and cultured in HHStec medium (Innoprot; Catalog# P60126) containing 10% FBS, 100 U/L penicillin, and 100 g/L streptomycin.

Cell treatment. Cells were plated onto 6-well plates. Before treatment, cells were rinsed with PBS, and added 1 ml new culture medium without FBS for each well. Inhibitors were pretreated for 1 h or 30 min, followed by an addition of UDCA-LPE to a final 90 μM. Control cells were treated with an equal volume of solvent.

Western blotting. After treatment, plates with cells were rinsed twice with cold PBS. Cells were lysed on ice with cell lysis buffer (20 mM Tris.HCl pH 7,4, 150 mM NaCl, 10 mM $MgCl_2$, 2 mM EDTA, 10% glycerol, 1% triton X-100, 2.5 mM β-glycerophosphate, 1 mM NaF, 1 mM DTT, 100 μg/ml PMSF, 1 mM sodium orthovanadate, 10 μl/ml protease inhibitor cocktail (Merck Millipore, Darmstadt, Germany). Lysates were obtained following centrifugation at 13,000 rpm for 10 min, and protein contents were determined by using Bradford Bio-Rad^{DC} kit (Bio-Rad Laboratories, Munich, Germany). Proteins in lysates were separated by SDS-PAGE gel electrophoresis and transferred onto PVDF membrane (Merck Millipore, Darmstadt, Germany). Blots were blocked in 5% milk containing TBST, and treated with a primary antibody overnight at 4°C. The membrane was washed and incubated with a secondary antibody for 1 h at room temperature. The immunoreactive signals were detected by an ECL detection system (Merck Millipore, Darmstadt, Germany).

Immunofluorescence. Cells were plated onto coverslips for 24 h. After treatment at indicated time, cells were washed three times with cold PBS and were fixed with 4% paraformaldehyde in PBS for 30 min. After rewashing 4 times for 5 min each time, cells were permeabilized in PBS containing 10% normal donkey serum (Santa Cruz Biotechnology, Heidelberg, Germany) and 0.3% Triton X-100 at room temperature for 1 h. Coverslips were incubated with a primary antibody overnight at 4°C. Coverslips were washed 3 times with PBS and incubated with a secondary antibody, phalloidin CruzFluor 488 Conjugate (Santa Cruz Biotechnology, Heidelberg, Germany), and DAPI at room temperature for 1 h. Phalloidin CruzFluor 488 Conjugate and DAPI were prepared in PBS containing

10% BSA and 0.3% Triton X-100 . Coverslips were washed five times in PBS, and then mounted in 50% PBS-50% glycerol.

Immunoprecipitation. Cells were lysed with RIPA Lysis Buffer (Santa Cruz Biotechnology, Heidelberg, Germany) and protein content was quantified by Bradford assay (Bio-Rad, CA, USA). Lysates at 500 µg protein were incubated with primary antibodies and protein A/G agarose beads (Santa Cruz Biotechnology, Heidelberg, Germany) at 4°C overnight. The samples were centrifuged at 12000 rpm for 15 min, and the pellet was washed for three times with RIPA lysis Buffer. The pellet was resuspended in 30 µl Laemmli sample buffer and incubated at 95°C for 5 min. The samples were centrifuged, and the supernatants were used for western blotting.

Supplementary Data

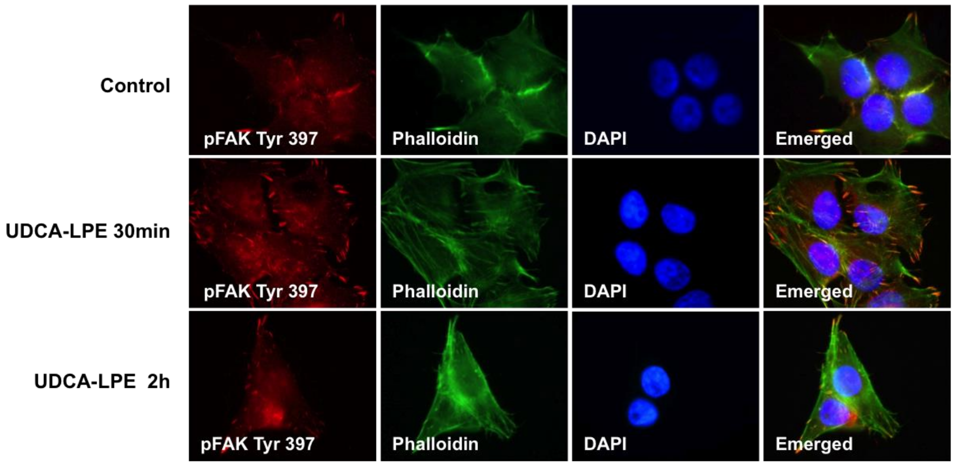

Figure S1. UDCA-LPE has no effect on the localization of pFAK Tyr397. Representative fluorescence microscopy images of CL48 cells after treatment with 90 µM UDCA-LPE for 30 min or 2h. Immunofluorescence staining of treated cells showed localization of pFAK Tyr 397 (red), FITC-phalloidin (green), and DAPI (blue). FITC-phalloidin was used for actin staining, and DAPI was used for nuclear staining.

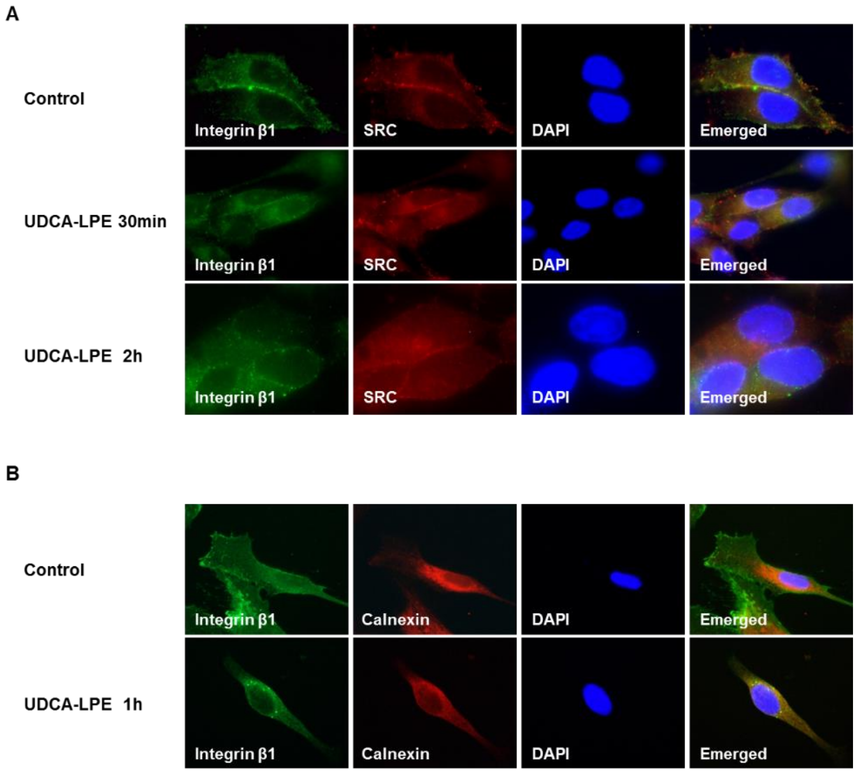

Figure S2. UDCA-LPE modulates the localization of integrin β1 in HHstec cells Representative fluorescence microscopy images of HHstec cells after treatment with 90 μM UDCA-LPE for (**A**) 30 min or 2 h and for (**B**) 1h. (**A**) Immunofluorescence staining of HHstec cells showed localization of integrin β1 (green), SRC (red) and DAPI (blue). (**B**) Immunofluorescence staining of HHstec cells showed localization of integrin β1 (green), calnexin (red) and DAPI (blue). DAPI was used for nuclear staining.

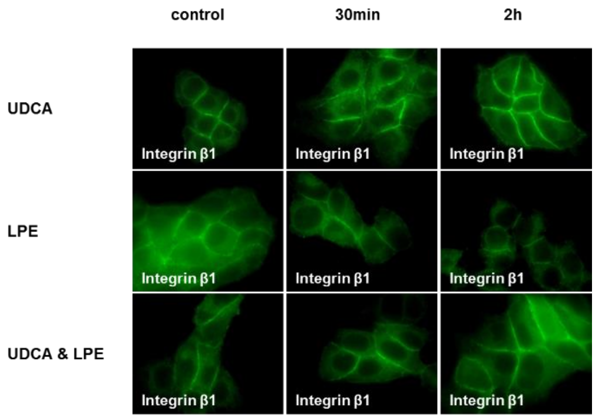

Figure S3. UDCA and LPE do not modulate the localization of integrin β1. Representative fluorescence microscopy images of CL48 cells after treatment with 90 μM UDCA, 90 μM LPE or 90 μM UDCA and 90 μM LPE for 2h. Pictures showed immunofluorescence staining of integrin β1 (green).

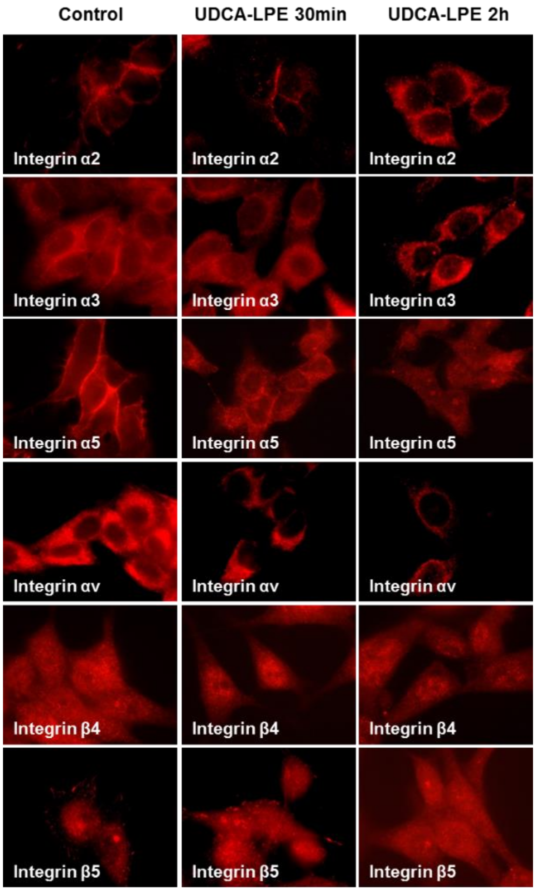

Figure S4. UDCA-LPE modulates the localization of integrins. Representative fluorescence microscopy images of (**A**) CL48 cells after treatment with 90 μM UDCA-LPE for 30min and 2h. Immunofluorescence staining of integrin α2, α3, α5, αv, β5, (**A**) β4 and (**B**) β3.

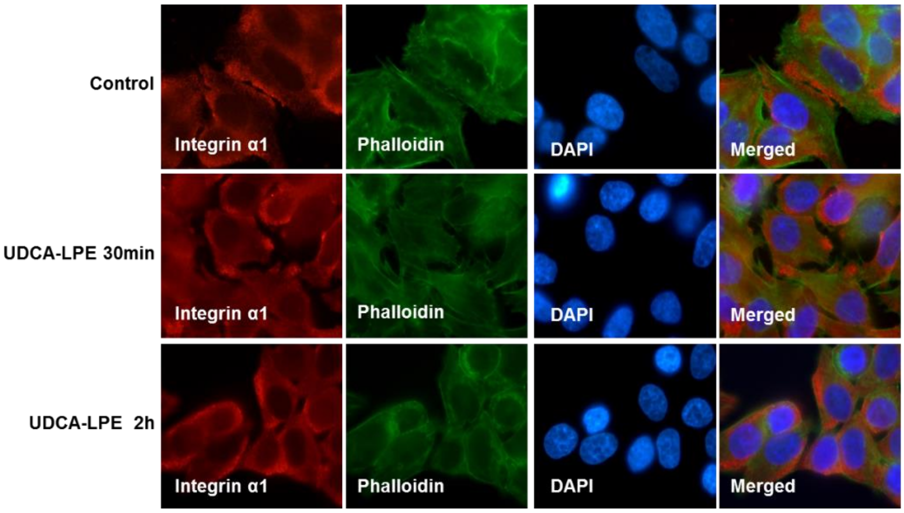

Figure S5. Lack of integrin α1 translocation by UDCA-LPE. Representative fluorescence microscopy images of CL48 cells after treatment with 90 μM UDCA-LPE for 30 min or 2 h. Immunofluorescence staining of integrin α1 (red), phalloidin CruzFluor 488 (green), and DAPI (blue). Phalloidin CruzFluor 488 was used for actin staining. DAPI was used for nuclear staining.

© 2018 by the authors. Submitted for possible open access publication under the terms and conditions of the Creative Commons Attribution (CC BY) license (http://creativecommons.org/licenses/by/4.0/).
